# 1013. Outpatient parenteral antimicrobial therapy (OPAT)-related peripheral eosinophilia as a predictor of hypersensitivity reactions.

**DOI:** 10.1093/ofid/ofac492.854

**Published:** 2022-12-15

**Authors:** Suha Abu Khalaf, Fahed Jubair, Nathanial S Nolan, Olujimi Jegede

**Affiliations:** University of Missouri health care, Columbia, Missouri; University of Jordan, Amman, 'Amman, Jordan; Washington University in St. Louis, St. Louis, Missouri; The University of Kansas Medical Center, Kansas City, Missouri

## Abstract

**Background:**

The administration of outpatient parenteral antimicrobial therapy (OPAT) has significantly increased in recent decades. OPAT offers many benefits to patients and the health care system. Best practices suggest patients on OPAT require active monitoring for adverse effects of therapy. Asymptomatic eosinophilia is common finding in patients receiving OPAT and could be benign or herald allergic drug reactions. Little is known about the practice patterns of OPAT providers in their management of asymptomatic eosinophilia. This study aimed to assess the opinions and practice patterns regarding asymptomatic eosinophilia among providers managing patients on OPAT in the United States.

**Methods:**

This is a cross-sectional study of data collected from a one-time, anonymous, self-administered survey to providers who care for patients receiving OPAT. The survey was posted in the discussion forums of the Infectious Diseases Society of America (IDSA) and shared via emails to twenty ID departments in the US. Data was collected from June to July 2021. Survey responses were analyzed using Python for statistical analysis.

**Results:**

Sixty-five respondents completed the survey. Thirty-six percent of respondents reported that eosinophilia occurs in up to five percent of their patients, with an average time of 1-2 weeks between antibiotic exposure and the development of eosinophilia Fig (1). Sixty-nine percent of respondents reported that they do not change their laboratory monitoring frequency in patients with asymptomatic eosinophilia. Fifty-two percent of respondents would discontinue/switch antibiotics only if the patient developed complications Fig (2). The risk of subsequent hypersensitivity reaction/end-organ damage was less frequently noted, as seventy-five percent encountered it in < 10% of eosinophilia cases Fig (1).

**Fig. 1:**
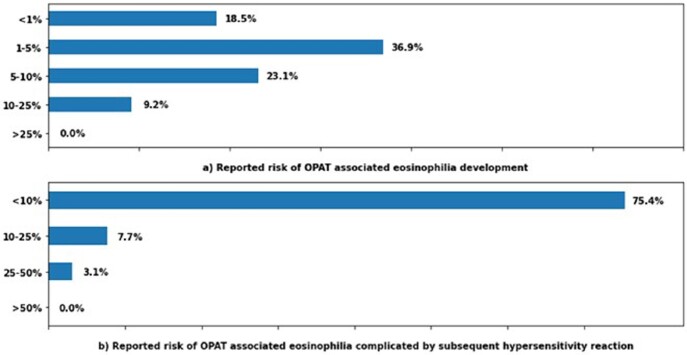
Reported OPAT associated eosinophilia and subsequent hypersensitivity reaction per respondents

**Fig. 2:**
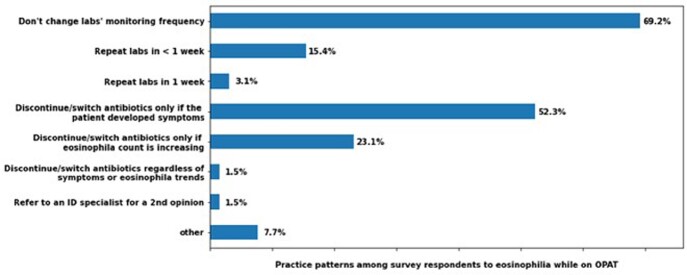
Practice patterns among survey respondents to eosinophilia

**Conclusion:**

Per our survey results, OPAT-related eosinophilia is common though subsequent hypersensitivity/life-threatening reactions are not. Most surveyed ID providers monitor asymptomatic eosinophilia without changing antibiotics. Further prospective studies are needed to understand the course and best practice for asymptomatic eosinophilia among patients on OPAT.

**Disclosures:**

**All Authors**: No reported disclosures.

